# Creation of a Global Vaccine Risk Index

**DOI:** 10.1371/journal.pone.0272784

**Published:** 2022-08-24

**Authors:** Tasmiah Nuzhath, Peter J. Hotez, Ashish Damania, P. Shuling Liu, Brian Colwell

**Affiliations:** 1 Department of Health Behavior, Texas A&M School of Public Health, College Station, Texas, United States of America; 2 Hagler Institute for Advanced Study at Texas A&M University, College Station, Texas, United States of America; 3 Texas Children’s Hospital Center for Vaccine Development and Center for Medical Ethics and Health Policy, Departments of Pediatrics and Molecular Virology & Microbiology, National School of Tropical Medicine, Baylor College of Medicine, Houston, Texas, United States of America; 4 Scowcroft Institute of International Affairs, Bush School of Policy and Government, Texas A&M University, College Station, Texas, United States of America; 5 Department of Biology, Baylor University, Waco, Texas, United States of America; 6 James A Baker III Institute of Public Policy, Rice University Houston, Houston, Texas, United States of America; 7 M.D. Anderson Cancer Center, Houston, Texas, United States of America; 8 Statistical Collaboration Center, Department of Statistics, Texas A&M University, College Station, Texas, United States of America; Public Health England, UNITED KINGDOM

## Abstract

The World Health Organization has identified vaccine hesitancy as one of its top ten global health threats for 2019. Efforts are underway to define the factors responsible for reductions in vaccine confidence. However, as global measles cases accelerated beginning in 2018, it became evident that additional factors were promoting measles re-emergence, including war, political and socio-economic collapse, shifting poverty, and vulnerability to weather events and climate change. Accordingly, we propose a Global Vaccine Risk Index (VRI) to consider these variables as a more comprehensive means to identify vulnerable nations where we might expect measles and other vaccine-preventable diseases to emerge or re-emerge. In Sub-Saharan African and Middle Eastern nations, conflict and political instability predominated as the basis for high vaccine risk scores, whereas in Southeast Asian countries, the major reasons included climate variability, current levels of measles vaccination coverage, and economic and educational disparities. In Europe, low vaccine confidence and refugee movements predominated, while in the Americas, economic disparities and vaccine confidence were important. The VRI may serve as a useful indicator and predictor for international agencies committed to childhood immunizations and might find relevance for accelerating future COVID19 vaccination programs.

## Introduction

In the years prior to the current COVID-19 pandemic, there was also a sudden and unexpected rise in the number of global measles cases and deaths. In 2018, the World Health Organization (WHO) and US Centers for Disease Control and Prevention (CDC) jointly reported 140,000 measles deaths, an increase from the year before [1], while during the first three months of 2019, the WHO found that reported measles cases increased by 300% relative to that same period in 2018 [2]. A rise in measles has special significance because it is typically the first childhood infectious disease to return following declines in vaccine coverage, a consequence of the high transmissibility of the measles virus [3]. Therefore, the re-emergence of measles can signal an interruption in vaccination programs for a variety of reasons, including social turmoil due to conflict, poverty that precludes access to vaccines or health care, or “vaccine hesitancy” which refers to the delay or refusal of parents to vaccinate their children despite the availability of the vaccine [4, 5]. Indeed, in 2019 the WHO listed vaccine hesitancy as a leading global threat [6].

The global return of measles also represents a public health setback that follows almost two decades of successes in global vaccination programs beginning with the launch of Gavi, the Vaccine Alliance, in 2000 [1]. At least a dozen nations have experienced measles outbreaks over the last three years, while others have sustained prolonged measles transmission. The reasons for this require further investigation, but so far, large outbreaks have occurred in selected countries across almost every continent. Measles outbreaks have recently occurred in multiple Sub-Saharan African nations, including the Democratic Republic of the Congo, Ethiopia, Nigeria, Tanzania and Madagascar; South and Southeast Asian countries, including Pakistan, India, Bangladesh, Myanmar, Philippines and Thailand; and in Europe and post-Soviet states, including Georgia, Kazakhstan, Kyrgyzstan, and Ukraine [2]. In addition, measles epidemics occurred in the Middle East and Central Asia [7], Venezuela and neighboring Brazil and Colombia [8], and in the United States [9].

In order to understand the basis for why measles and other vaccine-preventable diseases have returned, Larson et al. established a landmark vaccine confidence indicator [10, 11]. Vaccine confidence alone, however, may not explain why measles has returned in areas of conflict, such as in the Democratic Republic of the Congo; areas of political collapse such as in Venezuela or Myanmar; or in areas with extreme poverty and overall failures in health systems [5]. Barriers to vaccination coverage include demographic, socio-economic, geographic, cultural, and political and religious factors [12]. In addition to these determinants, environmental and socio-structural factors contribute to lower immunization rate.

Several studies have found that low educational levels and low socioeconomic status of parents, particularly mothers, impact adherence to vaccination regimens. Evidence from low-income countries suggests that mother’s education improves awareness related to health behaviors, which in turn has been showed to play an important role in the use of health care services, including immunization of children [13–15]. Lack of education and awareness also present a challenge to vaccine uptake as it leads to misconceptions or misinformation about vaccines and it negatively contributes to measles vaccination uptake [16].

Climate change has resuted in increased extreme weather events, contributing to disease outbreaks, including measles [17]. Additionally, disruption in healthcare services such as immunization due to climate change-driven extreme weather events can also result in outbreaks of vaccine preventable diseases (VPD). Climate change, as well as poverty and income disparities, has also resulted in both internal and cross border displacement of populations [18–20].

A decline in vaccination coverage is further exacerbated by a weakening of public health infrastructure. Millions of unvaccinated children live disproportionately in countries beset by conflict or in fragile states where ongoing conflict and hostilities collapse both health systems and vaccine delivery infrastructure [21, 22]. When hospitals and health facilities are damaged or destroyed, and when health workers are unable to do their jobs, it interrupts routine vaccination services. And in crowded camps and settlements, diseases like measles can spread rapidly. More than 95% of deaths from measles are in low income countries with weak health infrastructures [23].

Now in its second year, the global COVID-19 pandemic threatens vaccination systems. Health systems are strained not only by influxes of desperately ill people, but immunization systems are strained in ways never before seen as fforts to deliver vaccines to individuals worldwide, although such efforts are lagging in the poorest countries with the most fragile health systems. Beyond the social disruptions resulting from the pandemic there were concerns that the social determinants highlighted above, in addition to climate change, might prevent the full recovery of childhood vaccinaton programs. Therefore, there is a need to examine declines in vaccine coverage and the return of measles more broadly by including some of these additional determinants.

Herein we report on the development of a preliminary vaccine risk index and accompanying heat map, which incorporates published vaccine confidence estimates, in addition to indices of human development, conflict, disasters and refugee movements, and climate change metrics [21]. We find that such a Vaccine Risk Index reflects or approximates the geographic distribution of risk of measles outbreaks We explored the physical and social determinants responsible for the variation in measles vaccination coverage across countries to calculate a final VRI. Nations with high VRI estimates are those considered at greatest risk for breakthrough measles infections. They represent an effort to rank nations for vaccine risk along the lines of the social and physical determinants proposed previously [5].

## Methods

### Data

We considered a variety of influences related to vaccine confidence, climate change, human development, conflict and instability, and other social determinants and obtained data for 150 countries to examine factors influencing measles immunization uptake. Countries were grouped into six world regions according to WHO regions: Africa, the Americas, the Eastern Mediterranean, Europe, South-East Asia, and the Western Pacific.

We obtained data for our initial list of variables from a variety of sources for 2017 and 2018. A summary of the eleven sources used or eliminated to derive a composite Vaccine Risk Index and risk map are summarized in Table 1.

**Table 1:** Sources for the variables and parameters considered in assessing a Vaccine Risk Index.

Data	Year	Included in Final index	Source URL
Vaccine confidence index (Net Vaccine confidence percent)	2018	Yes	https://wellcome.ac.uk/sites/default/files/wgm2018-dataset-crosstabs-all-countries.xlsx [24]
ND-GAIN index value	2017	No (Spearman correlation > 0.70 with HDI)	https://gain.nd.edu/our-work/country-index/download-data/ [25]
Climate risk index	2017	Yes	https://germanwatch.org/sites/germanwatch.org/files/Global%20Climate%20Risk%20Index%202019_2.pdf https://germanwatch.org/en/16046 [26]
HDI index value	2017	Yes	http://hdr.undp.org/en/data [27]
Measles incidence rate per 100,000 (Ages 1 to 4)	2017	Yes	http://ghdx.healthdata.org/gbd-results-tool?params=gbd-api-2017-permalink/dc4ba95a2666c749ed58ab8ff0094475 [28]
Measles Vaccine Coverage (MCV1 coverage)	2017	Yes	https://www.who.int/immunization/monitoring_surveillance/data/en/ http://www.who.int/entity/immunization/monitoring_surveillance/data/coverage_series.xls (MCV1) coverage data for 2017 were downloaded from the WHO [29].
Percent population internally displaced by a disaster	2017	No (Missing data > 30%)	https://data.worldbank.org/indicator/VC.IDP.NWDS [30]
Peace Index	2017	Yes	http://visionofhumanity.org/app/uploads/2017/06/GPI17-Report.pdf [31]
Urban Population percent	2017	No (Spearman correlation value > 0.70 with HDI)	https://data.worldbank.org/indicator/SP.URB.TOTL.IN.ZS [32]
Refugee population by country of origin	2017	Yes	https://data.worldbank.org/indicator/SM.POP.REFG.OR [33]
Percent population internally displaced due to conflict and violence	2017	No (Missing data > 30%)	https://data.worldbank.org/indicator/VC.IDP.NWCV [34]

### Variables

**Fig 1:**
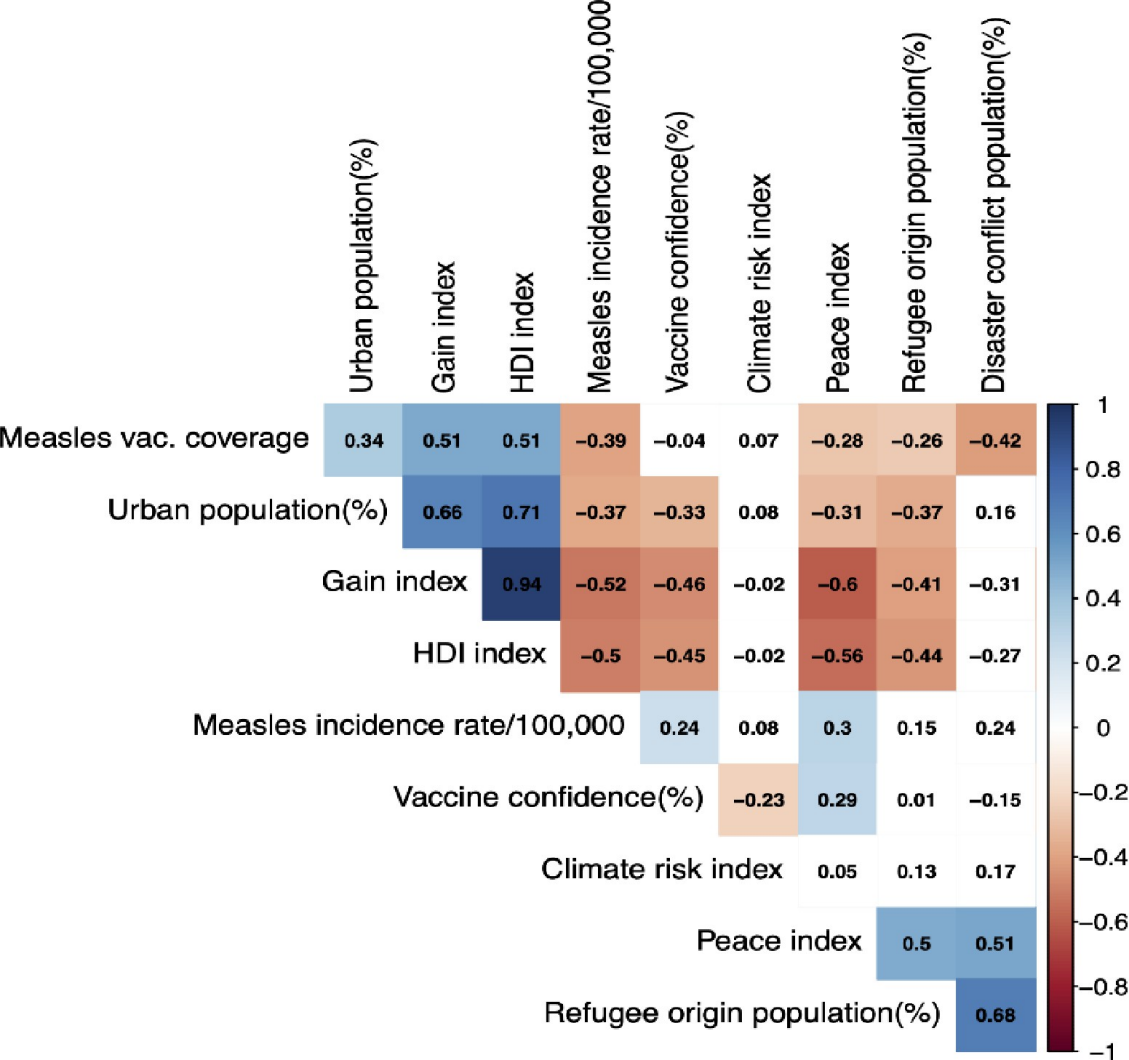
Spearman correlations between the variables involved in derivation of VRI. The colored vertical bar shows Spearman’s correlation between the variables with the intensity of color indicating the increasing absolute correlation. Red and blue areas in the bar correspond to <0 to-1 and> 0 to 1 Spearman correlation values, respectively.

From eleven potential variables, the final seven were selected based on the following considerations: (1) variables with more than 30% missing values were excluded from further analysis; (2) Pairwise spearman correlation coefficients were calculated as shown in Fig 1. In the two cases in which there was a correlation of variables over 0.7 with the human development index they were considered redundant and therefore discarded.

### Sources of data for selected variables

- We selected a component of vaccine confidence based on the question, “Do you strongly agree, agree, neither agree nor disagree, disagree or strongly disagree with the following statement? “Vaccines are safe” using data from the 2018 Wellcome Global Monitor [11]. Our vaccine confidence value for each country was calculated by subtracting the percentage of people disagreeing or strongly disagreeing with the question above from those agreeing or strongly agreeing.
- The University of Notre Dame Global Adaptation Index (ND-GAIN), that ranks climate adaptation [12], incorporating climate risk index data that were obtained from Germanwatch.org [13].
- Urban population percentage, refugee population by origin country, percent population internally displaced due to conflict and violence, and percent population internally displaced due to disaster were obtained from the World Bank.
- The peace index was acquired from Vision of Humanity: http://visionofhumanity.org/indexes/global-peace-index/.
- Human development index data, which measures overall achievement in several economic and social dimensions, were selected and downloaded from the United Nations Development Programme (UNDP) [14].
- Finally, the measles incidence rate per 100,000 for each country was obtained from the Institute for Health Metrics and Evaluation (IHME) [15].

### Normalization of variables

**Fig 2:**
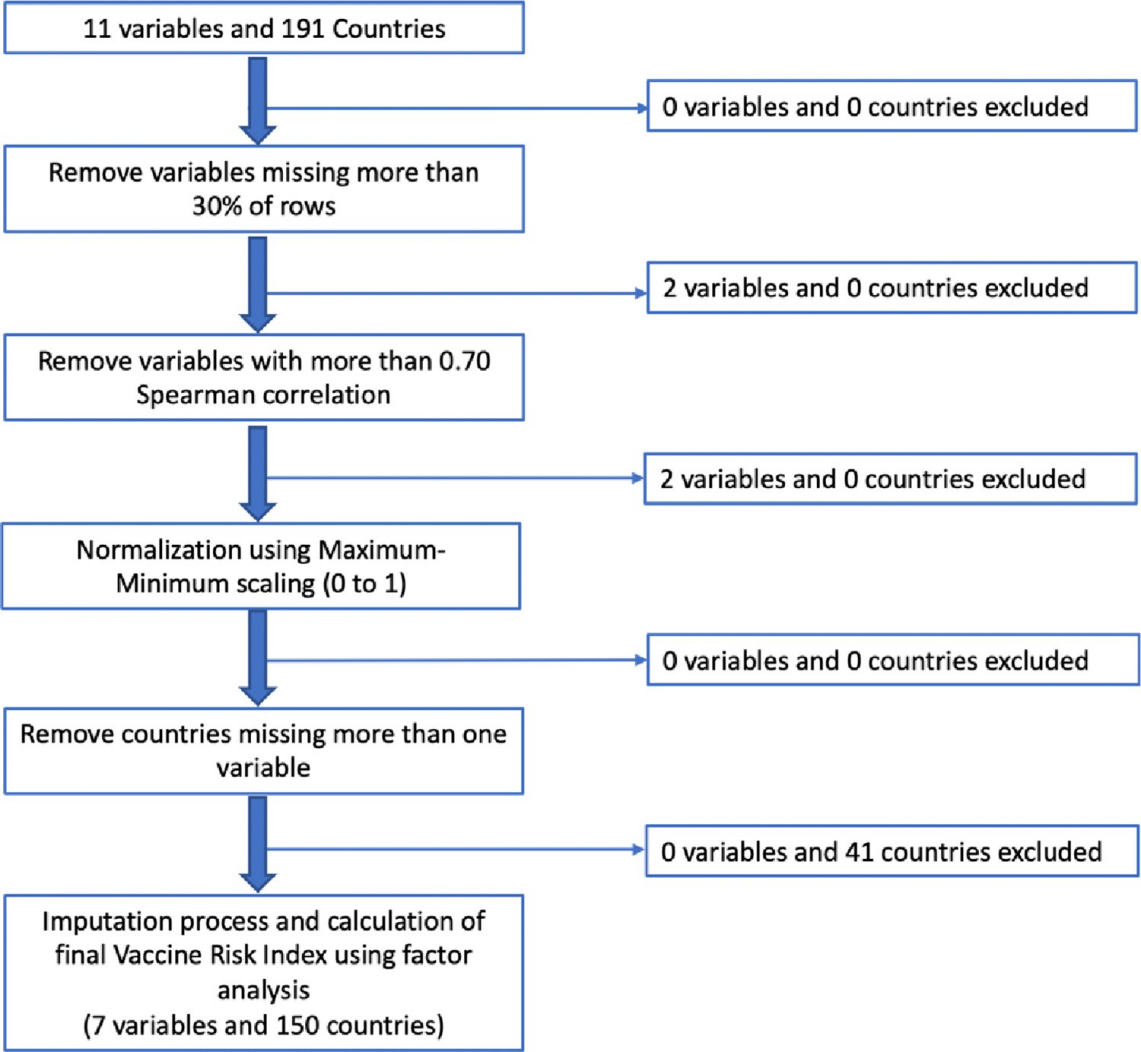
Steps in creating the VRI. The boxes show the initial number of variables and intermediate steps to filter the variables and countries for the final vaccine risk index factor analysis calculation.

Each of the included variables was scaled from 0 to 1 using min-max normalization to account for scaling differences between variables related to peace and climate risk indices. Certain variables such as measles containing vaccine dose 1 (MCV1) percent coverage, Climate risk index, HDI index, and overall vaccine confidence were expressed to reflect that lower values indicate better outcomes. Countries missing more than one variable were removed from the analysis, and missing data were subsequently imputed. Fig 2 shows the procedure undertaken to calcute the VRI scores.

### Vaccine Risk Index score development

The vaccine risk index (VRI) was calculated as follows: We first used factor analysis to determine how the variables clustered, which was performed via maximum likelihood factoring with oblique rotation to a final solution. All eigenvalues in the final solution exceeded 1. The first factor includes measles incidence and vaccine coverage statistics. The second factor is based on vaccination confidence. All of the items in the third factor are related to war and conflict. The fourth factor relates to climate change, while the fifth factor includes items related to education and income. Table 2 includes the variance explained by each factor.

**Table 2:** Final solution with five factors (Ordered by loading).

	Vaccinations	Vaccine Confidence	Conflict	Climate	Education & Poverty
Variables	Measles Incidence rate (0.6)	Vaccine confidence index (0.8)	Peace Index Score(0.7)	Climate risk Index (0.4)	Human Development Index (0.4)
	MCV1 percentage coverage (0.8)		Percentage refugee population (0.6)		
% of variance explained	34.91%	23.31%	26.37%	7.94%	7.47%

The resulting output from the factor analysis was used to determine factor scores. The final VRI value was calculated by summing all of the factor scores from the previous steps. Higher VRI values denote the highest risk and lower values indicate the lowest at-risk status. The R programming code for the data processing and figures is available at https://github.com/ashishdamania/vaccine_risk_index.

**Fig 3:**
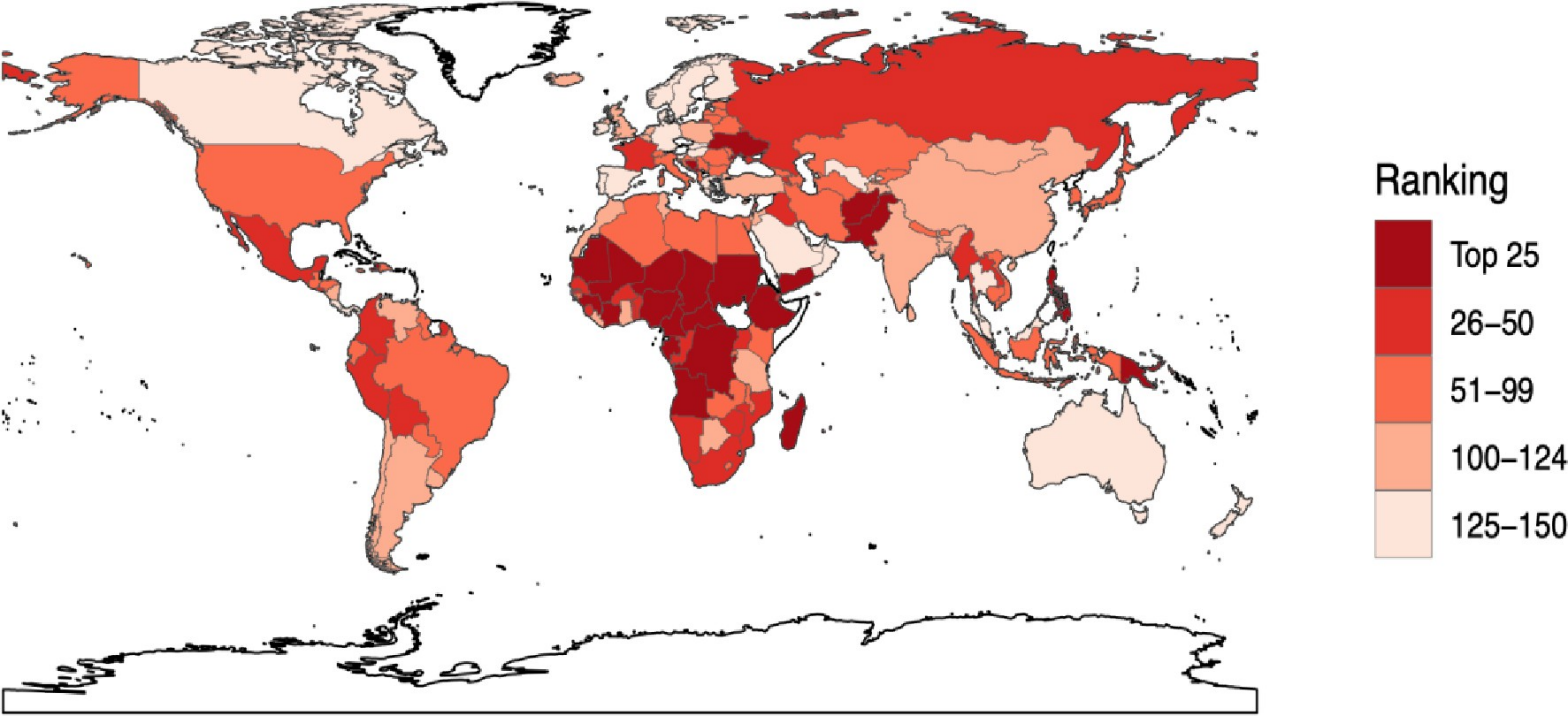
Heat map of Vaccination Risk Indices. Darker colors indicate a higher VRI score, indicating greater outbreak risk. *Generated using public domain map data from https://www.naturalearthdata.com/.

Fig 3 is a visual heat map comparing nations with respect to different tiers of the VRI. It demonstrates that the top 25 at-risk countries are predominantly in Sub-Saharan Africa, in addition to Yemen in the Middle East, Afghanistan, Sudan and Pakistan in the Eastern Mediterranean region, Papua New Guinea and Philippines in the Western Pacific region, and Ukraine in Europe. A majority of the lowest-risk 25 nations are in Europe and the Americas, although Mexico, Colombia, Peru, and Bolivia were relatively high. All VRI scores are included in S1 Appendix.

## Results

### Major findings

In Europe, Montenegro, Bosnia and Herzegovina, Ukraine, Russian Federation and France are among the high-risk countries. Ukraine and Russia, beset by conflict, also demonstrate high VRI scores. These findings are similar to the WHO’s list of countries with significant measles outbreaks in the European region [24]. Countries in Europe with low VRI scores (less risk) include Norway, Hungary, Portugal, Denmark, Spain, and Sweden.

In the Americas, Haiti, Peru, Mexico and Bolivia are among the leading vaccine risk nations with high VRIs, although the high amount of transnational migration elevates the risk for much of the region. In the same region, low risk countries include Panama, Canada and Costa Rica.

In the WHO Western Pacific Region, Papua New Guinea, the Philippines, Laos and Cambodia are at high risk. Conversely, Australia, Singapore and New Zealand exhibit overall low VRI scores.

In Africa, the Central African Republic has the highest VRI score as do other several key conflict-associated and low-resource countries including Chad, Mali, Equatorial Guinea, DR Congo, Madagascar, Angola, Cote d’Ivoire, and Gabon. In contrast, nations such as Botswana, Mauritius, and Zambia are at lower risk. In the Middle East and the WHO Eastern Mediterranean region, Afghanistan, Yemen, Pakistan, Iraq and Sudan are the nations of greatest concern, overlapping with WHO measles reported cases [24]. By contrast, Qatar, United Arab Emirates, Kuwait, and Oman have lowest VRI scores in this region. Indonesia, Myanmar, Timor Leste and Bhutan are among countries at highest risk in the WHO South-East Asia region, whereas Sri Lanka and Thailand have an overall lowest risk.

### Factor component analysis

**Fig 4:**
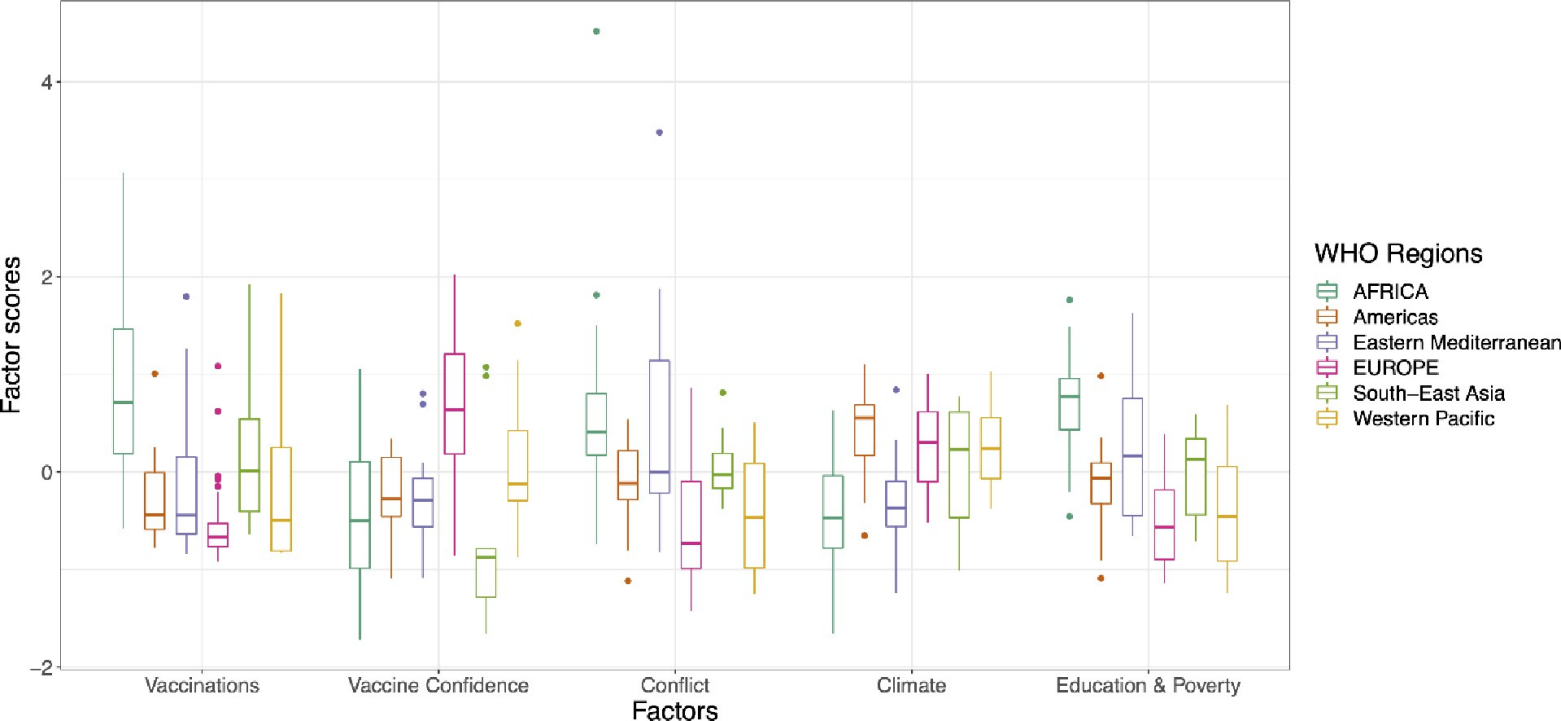
Factor score distributions by WHO Regions.

The boxplot in Fig 4 provides a factor component analysis and illustrates the distribution of factor scores across the WHO regions. The factor scores indicate the contribution of the components towards overall risk for each region. The boxplots for each factor region-wise are uneven, demonstrating the different contributions of various components in each region. These regional variations are caused by significant differences between individual countries Fig 4. Factor score distributions by WHO Regions.

#### Africa

The countries in African region with high VRIs have a high measles incidence rate as reflected in a high vaccinations factor (low coverage) score, and war and political instability as characterized by a low peace index and large refugee populations (conflict factor score). In contrast, countries with low VRI are peaceful and comparatively affluent nations, with high measles vaccination coverage and high vaccination confidence indices that contribute to their overall low risk index.

#### The americas

Climate change is the major contributor for high VRIs in the Americas, in assocation with low vaccination coverage, high measles incidence, and low vaccination confidence, in addition to significant education and wealth disparities. In comparison, countries in the Americas that exhibited high measles vaccination coverage and high vaccination confidence along with high HDI score, have low VRIs.

#### Middle east

In the Middle East and the greater WHO Eastern Mediterranean region, the major driver for high VRI is war or conflict (driven by the conflict factor score). In comparison, countries in this region with high educational attainment, economic opportunity and political stability contribute to low risk indices.

#### Europe

In Europe, the major contributors to a high vaccine risk index are lower vaccine confidence index and high climate risk as reflected in high vaccine confidence and climate factor scores. In contrast, risk is low in countries with lower measles incidence rate, low peace index scores and low education and wealth disparities.

#### South Asia

Despite having high vaccination confidence, countries at highest risk in the WHO South-East Asia region were characterized by low immunization coverage due to vulnerability to climate variability, economic and educational disparities, and a high rate of measles incidence. Countries in this region with high HDI, and high measles vaccination coverage by contrast, have an overall low risk.

#### Western pacific

In the WHO Western Pacific Region, a high Vaccinations factor score, a high measles incidence rate, and low vaccination confidence, contribute to putting the populations of these countries at high risk. Conversely, countries that exhibited less education and economic disparities and low peace index scores resulted in an overall low vaccination risk index.

Table 3 provides additional granular detail by summarizing and comparing nations with high VRI values with the WHO’s recent listing of nations with the highest incidence of measles infections. Of the 20 nations with the highest incidence rates of measles infections in 2019, five of them–Madagascar, Ukraine, Central African Republic, Yemen, and Democratic Republic of the Congo–exhibited high VRI scores [35]. These nations regularly appear on the list of most measles cases although the order varies by the size and recency of outbreaks. A difference between the top 20 VRI scores and the top 20 reported measles incidence scores indicates that the VRI scale is indeed qualitatively different, and taps into a broader array of determinants, than the nations with the most cases of measles.

**Table 3:** Top 20 vaccine risk nations and WHO’s 2019 list of the top 20 most measles endemic nations.

Rank	Rank and Nation by Vaccine Risk Index	VRI Score	Rank & Nation by Measles incidence rate (WHO) for the Year 2019	Rank & Nation by Reported Measles Case (WHO) for 2019
1	Central African Republic*	8.87	Madagascar*	Madagascar*
2	Afghanistan	6.01	Ukraine*	Ukraine*
3	Chad	5.74	Georgia	Philippines
4	Guinea	4.22	The Republic of North Macedonia	Nigeria
5	Yemen*	4.17	Kazakhstan	Brazil
6	Mali	3.96	New Zealand	DR Congo*
7	Haiti	3.37	Philippines	Yemen*
8	Montenegro	3.33	Bosnia and Herzegovina	Kazaksthan
9	Angola	3.30	Tunisia	India
10	Ukraine*	2.92	Central African Republic*	Bangladesh
11	DR Congo*	2.83	Kyrgyzstan	Thailand
12	Papua New Guinea	2.80	San Marino	Myanmar
13	Ethiopia	2.40	Yemen*	Vietnam
14	Madagascar*	2.37	Tonga	Russian Federation
15	Nigeria	2.33	Lithuania	Georgia
16	Mauritania	2.30	Samoa	Ethiopia
17	Pakistan	2.29	DR Congo*	Sudan*
18	Cote d’Ivoire	2.21	Liberia	China
19	Sudan*	2.03	Bulgaria	Angola
20	Gabon	1.98	Lebanon	Turkey

Source: WHO. Global Measles and Rubella monthly update [24]

*Nations ranked in at least two columns.

Our list of nations with a high VRI differs somewhat from the WHO list of nations with high measles incidence, indicating a greater level of granularity in the VRI than merely examining measles indicence. For example, Ukraine is experiencing conflict and has a low vaccination confidence index, both of which contribute to its high VRI scores. This is similar to the WHO’s list of countries with significant measles outbreaks in the European region, but the actual causes are explained in the VRI score [35].

## Discussion

Our study undertook a global analysis exploring various social and physical determinants to create a Vaccine Risk Index. Although prior indices examine vaccination performance indicators [36] and vaccination confidence [10] across multiple countries, the VRI represents an effort to broaden the criteria in that it includes additional key social and physical determinants of particular relevance to the last decade. These include war, political instability, climate change and adaptation, and poverty, and human development indices. Such factors were hypothesized previously to be responsible for the return of vaccine-preventable and other diseases [5]. Overall, the majority of measles deaths occur in countries with low per capita income and fragile healthcare systems [23], and the VRI, while including MCV1 coverage and measles incidence, may be a better predictor of outbreaks of infectious disease because it includes those additional social and physical determinants.

The major drivers of high VRI values also varied by region. For instance, in Africa and the Middle East, armed conflict and political instability as well forced displacement were dominant, whereas in Asian countries, a low human development index value and climate variability were key drivers. While it is important to note that this instrument does not simply mirror existing measles prevalence and outbreak statistics, the results demonstrate alignment with previous studies and verify the importance of the role that socio-economic and environmental factors play in observed levels of vaccination coverage [23, 37–39].

Alternatively, many of the nations in other parts of the world with high VRIs, such as Sudan, Central African Republic, Chad, Afghanistan, and Equatorial Guinea are characterized by armed conflict, which likely results in either poor detection or underreporting of measles case data but also leads to reduced opportunities to access vaccines of all types. In these countries, national conflict has collapsed healthcare and vaccine logistics, depleted human resources, and decreased access to immunization services, producing pockets of low vaccination coverage.

Outside of the conflict areas in Africa and the Middle East, or impoverished nations in Asia, in Europe, low vaccination confidence has resulted in limited vaccination coverage, while in the Americas both low HDI and vaccination confidence have been the driving factors. Our study results are aligned with the findings from previous studies [10, 40] that identified challenges in maintaining 95% vaccination coverage due to negative vaccination sentiment despite widespread accessibility to vaccination. To mitigate the risks associated with potential measles outbreaks, adequate context-specific measures can be designed and deployed.

### Limitations

This study had some limitations that also act as insights for further development of the study design. First, this study and its conclusions are limited by data availability. We restricted our analysis to include 150 countries only, and datasets for 2017 and 2018. Future versions of an index of this sort will be dependent on timely and accurate data collection of the data used herein. Additionally, differences in measurement techniques between surveys may contribute bias. Because of the lack of sub-national data for many of the indices used, the VRI did not account for differences within and between subnational regions, and it was not possible to stratify our results by urban and rural areas or in other more granular ways within countries.

The results of this study improve understandig of existing regional variability in correlates of vaccination coverage and adds important context to our metrics. Additional work is required to identify factors that have the potential to increase our understanding of how both high and low rates of vaccination coverage can exist in the same geographical areas so that appropriate policies and vaccination strategies can be identified.

## Supporting information

S1 Appendix (DOCX)

S1 File (DOCX)
