# Supplementary material for: Creation of a Global Vaccine Risk Index
Source: PLoS One. 2022 Aug 24;17(8):e0272784. doi: 10.1371/journal.pone.0272784 (PMC9401103; doi:10.1371/journal.pone.0272784)
Supplement: S1 Appendix — (DOCX) [file pone.0272784.s001.docx]

**Appendix A**

- - - Data Availability: All data used in the analysis were obtained from publicly available datasets.

The minimal dataset is located at: [https://dx.doi.org/10.6084/m9.figshare.17044568](https://urldefense.com/v3/__https:/dx.doi.org/10.6084/m9.figshare.17044568__;!!KwNVnqRv!UqL--fuzVfDQfUk8LdveiqQ7goeQtUyTJgi7drdS8e4TXWznj4--__H8dQe5Tsec6A$)

Final VRI scores for all nations

| Rank | Continent | Country | Final VRI Score |
| --- | --- | --- | --- |
| 1 | Africa | Central African Republic | 8.995 |
| 2 | Asia | Afghanistan | 6.085 |
| 3 | Africa | Chad | 5.827 |
| 4 | Africa | Guinea | 4.292 |
| 5 | Asia | Yemen | 4.180 |
| 6 | Africa | Mali | 3.970 |
| 7 | Africa | Angola | 3.447 |
| 8 | North America | Haiti | 3.409 |
| 9 | Europe | Montenegro | 3.378 |
| 10 | Europe | Ukraine | 2.875 |
| 11 | Australia | Papua New Guinea | 2.871 |
| 12 | Africa | Democratic Republic of the Congo | 2.823 |
| 13 | Africa | Madagascar | 2.492 |
| 14 | Africa | Ethiopia | 2.458 |
| 15 | Africa | Nigeria | 2.395 |
| 16 | Asia | Pakistan | 2.308 |
| 17 | Africa | Mauritania | 2.300 |
| 18 | Africa | CÃ´te d'Ivoire | 2.249 |
| 19 | Africa | Gabon | 2.069 |
| 20 | Europe | Bosnia and Herzegovina | 2.004 |
| 21 | Africa | Sudan | 1.994 |
| 22 | Africa | Cameroon | 1.932 |
| 23 | Asia | Philippines | 1.874 |
| 24 | Africa | Burundi | 1.845 |
| 25 | Africa | South Africa | 1.766 |
| 26 | Africa | Niger | 1.720 |
| 27 | Asia | Timor-Leste | 1.695 |
| 28 | Africa | Congo | 1.659 |
| 29 | Africa | Benin | 1.639 |
| 30 | Africa | Burkina Faso | 1.330 |
| 31 | Asia | Iraq | 1.283 |
| 32 | Africa | Togo | 1.237 |
| 33 | Europe | Russian Federation | 1.206 |
| 34 | North America | Mexico | 1.130 |
| 35 | Asia | Myanmar | 1.113 |
| 36 | Africa | Sierra Leone | 1.097 |
| 37 | South America | Peru | 1.050 |
| 38 | Africa | Djibouti | 0.948 |
| 39 | Africa | Uganda | 0.904 |
| 40 | South America | Bolivia (Plurinational State of) | 0.897 |
| 41 | Asia | Lao People's Democratic Republic | 0.859 |
| 42 | North America | El Salvador | 0.742 |
| 43 | Europe | France | 0.647 |
| 44 | Asia | Armenia | 0.622 |
| 45 | Africa | Mozambique | 0.599 |
| 46 | Africa | Senegal | 0.493 |
| 47 | Africa | Namibia | 0.453 |
| 48 | Asia | Lebanon | 0.395 |
| 49 | Europe | Serbia | 0.356 |
| 50 | South America | Colombia | 0.347 |
| 51 | Africa | Zimbabwe | 0.340 |
| 52 | Africa | Gambia | 0.306 |
| 53 | Asia | Cambodia | 0.292 |
| 54 | North America | Guatemala | 0.281 |
| 55 | South America | Ecuador | 0.245 |
| 56 | Europe | Belarus | 0.146 |
| 57 | Africa | Lesotho | 0.097 |
| 58 | Africa | Algeria | 0.058 |
| 59 | Europe | Bulgaria | 0.051 |
| 60 | Africa | Kenya | 0.029 |
| 61 | Asia | Kyrgyzstan | 0.023 |
| 62 | Africa | Libya | 0.002 |
| 63 | Africa | Malawi | -0.003 |
| 64 | Asia | Nepal | -0.111 |
| 65 | Europe | Croatia | -0.112 |
| 66 | Asia | Bhutan | -0.124 |
| 67 | Africa | Guinea-Bissau | -0.156 |
| 68 | Africa | Rwanda | -0.166 |
| 69 | Europe | Albania | -0.198 |
| 70 | Europe | Latvia | -0.226 |
| 71 | Africa | Comoros | -0.232 |
| 72 | Africa | Egypt | -0.241 |
| 73 | Europe | Romania | -0.243 |
| 74 | North America | Dominican Republic | -0.246 |
| 75 | Asia | Azerbaijan | -0.303 |
| 76 | Asia | Kazakhstan | -0.409 |
| 77 | Asia | Viet Nam | -0.414 |
| 78 | South America | Brazil | -0.437 |
| 79 | Europe | Cyprus | -0.463 |
| 80 | South America | Paraguay | -0.484 |
| 81 | Europe | Republic of Moldova | -0.496 |
| 82 | North America | United States of America | -0.519 |
| 83 | North America | Honduras | -0.588 |
| 84 | Europe | Estonia | -0.650 |
| 85 | Asia | Georgia | -0.696 |
| 86 | Asia | Indonesia | -0.714 |
| 87 | South America | Guyana | -0.740 |
| 88 | North America | Trinidad and Tobago | -0.742 |
| 89 | Asia | Japan | -0.820 |
| 90 | Asia | Iran (Islamic Republic of) | -0.822 |
| 91 | Africa | Mauritius | -0.828 |
| 92 | Africa | Zambia | -0.889 |
| 93 | Europe | Lithuania | -0.927 |
| 94 | Europe | Switzerland | -0.985 |
| 95 | Asia | Republic of Korea | -0.996 |
| 96 | Asia | Turkmenistan | -1.016 |
| 97 | Asia | Bahrain | -1.039 |
| 98 | Europe | Italy | -1.058 |
| 99 | Africa | Liberia | -1.059 |
| 100 | South America | Chile | -1.065 |
| 101 | South America | Argentina | -1.092 |
| 102 | Asia | Turkey | -1.093 |
| 103 | Asia | India | -1.094 |
| 104 | North America | Jamaica | -1.106 |
| 105 | Africa | Ghana | -1.127 |
| 106 | South America | Venezuela (Bolivarian Republic of) | -1.180 |
| 107 | Asia | Bangladesh | -1.210 |
| 108 | Asia | China | -1.224 |
| 109 | Asia | Mongolia | -1.237 |
| 110 | Europe | Belgium | -1.282 |
| 111 | Asia | Israel | -1.294 |
| 112 | Asia | Tajikistan | -1.305 |
| 113 | Europe | Netherlands | -1.373 |
| 114 | Asia | Sri Lanka | -1.402 |
| 115 | North America | Nicaragua | -1.441 |
| 116 | Africa | Botswana | -1.448 |
| 117 | Asia | Jordan | -1.465 |
| 118 | Europe | United Kingdom of Great Britain and Northern Ireland | -1.471 |
| 119 | South America | Uruguay | -1.492 |
| 120 | Europe | Poland | -1.530 |
| 121 | Africa | Tunisia | -1.544 |
| 122 | Africa | Morocco | -1.550 |
| 123 | Europe | Iceland | -1.552 |
| 124 | Asia | Thailand | -1.593 |
| 125 | Africa | United Republic of Tanzania | -1.604 |
| 126 | North America | Canada | -1.605 |
| 127 | Europe | Slovenia | -1.610 |
| 128 | North America | Costa Rica | -1.633 |
| 129 | Europe | Greece | -1.663 |
| 130 | Asia | Saudi Arabia | -1.686 |
| 131 | Europe | Slovakia | -1.735 |
| 132 | Europe | Germany | -1.760 |
| 133 | Europe | Ireland | -1.792 |
| 134 | Asia | Uzbekistan | -1.897 |
| 135 | Australia | New Zealand | -1.909 |
| 136 | North America | Panama | -1.951 |
| 137 | Asia | Qatar | -2.007 |
| 138 | Europe | Finland | -2.054 |
| 139 | Europe | Sweden | -2.085 |
| 140 | Asia | Malaysia | -2.159 |
| 141 | Europe | Spain | -2.189 |
| 142 | Asia | Oman | -2.211 |
| 143 | Australia | Australia | -2.249 |
| 144 | Europe | Portugal | -2.270 |
| 145 | Europe | Denmark | -2.403 |
| 146 | Asia | United Arab Emirates | -2.405 |
| 147 | Asia | Singapore | -2.430 |
| 148 | Asia | Kuwait | -2.432 |
| 149 | Europe | Hungary | -2.447 |
| 150 | Europe | Norway | -2.622 |
