# Supplementary material for: Creation of a Global Vaccine Risk Index
Source: PLoS One. 2022 Aug 24;17(8):e0272784. doi: 10.1371/journal.pone.0272784 (PMC9401103; doi:10.1371/journal.pone.0272784)

**Spearman correlations between the variables involved in the derivation of VRI.**


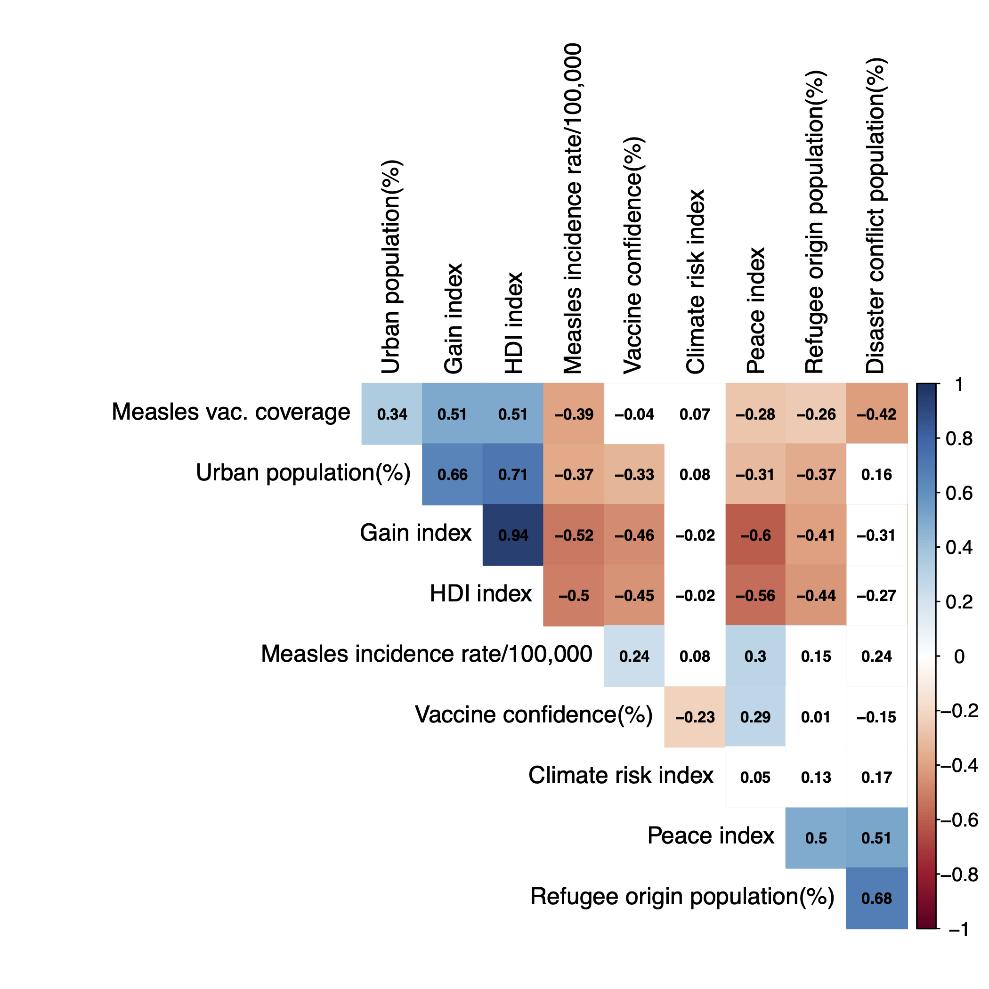


The colored vertical bar shows Spearman's correlation between two variables with the intensity of color indicating the increasing absolute correlation. Red and blue areas in the bar correspond to < 0 to -1 and > 0 to 1 Spearman correlation values, respectively.

**Steps in creating the VRI**


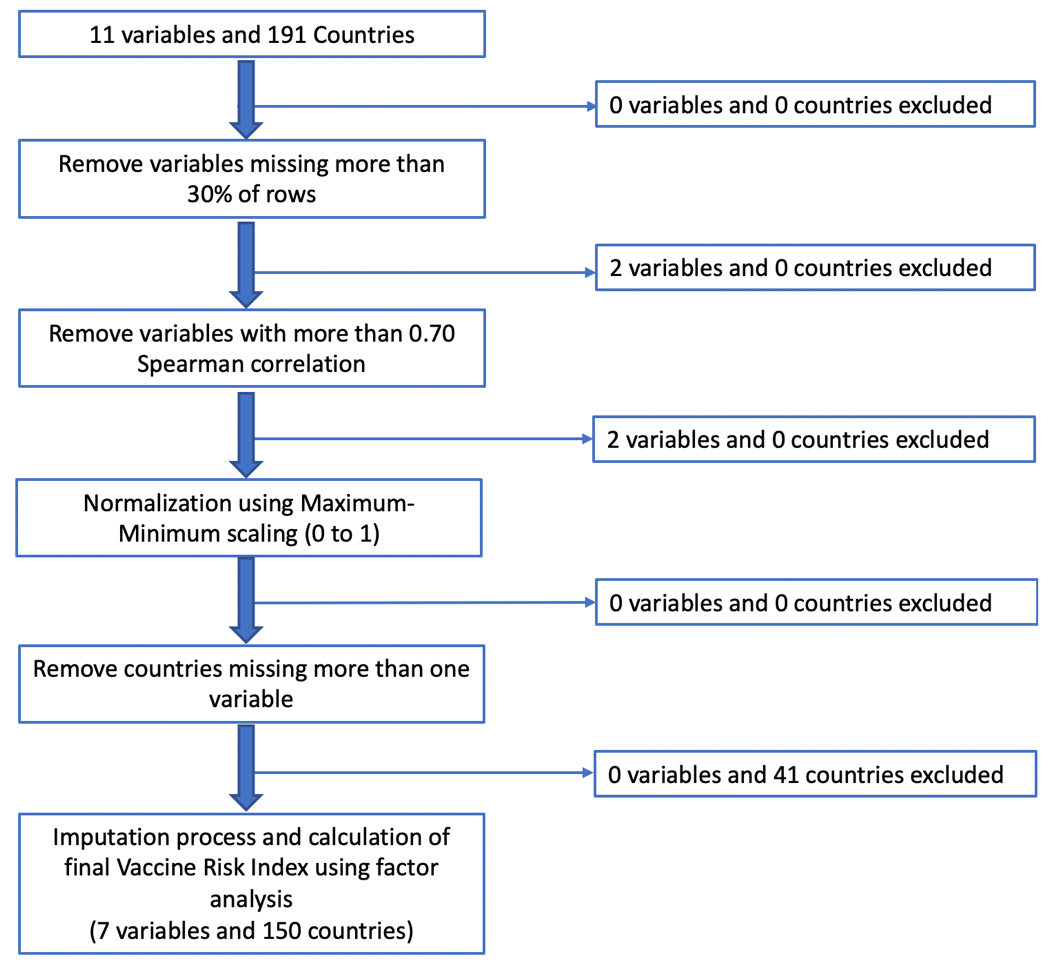


The boxes show the initial number of variables and intermediate steps to filter the variables and countries for the final vaccine Risk index factor analysis calculation

**Heat map* of Vaccination Risk Indices**


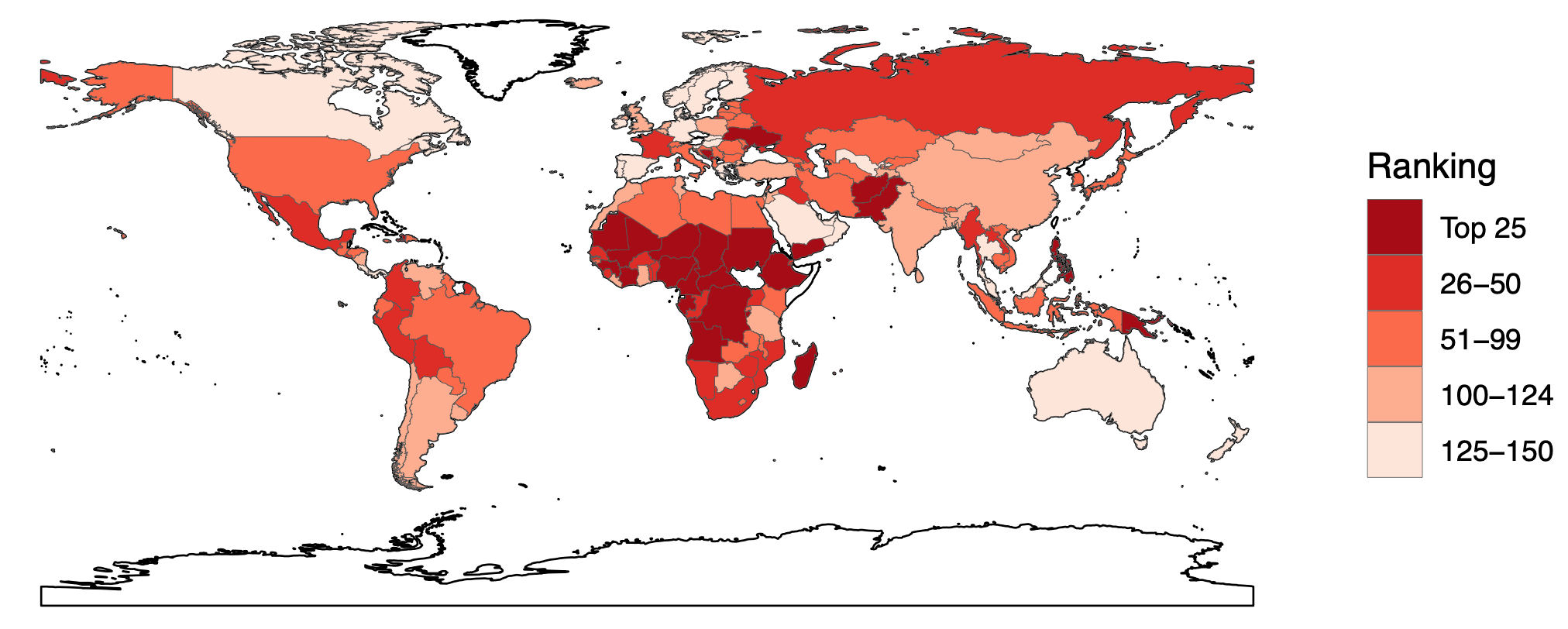


*Generated using public domain map data from [https://www.naturalearthdata.com/](https://urldefense.com/v3/__https:/www.naturalearthdata.com/__;!!KwNVnqRv!WeUjf-gOWXGxA1qqLJr0-s-IDYTB5s2Fzi2nYVuxzOmv75kkjjfseRMBEUh05-aQYQ$)

**Distribution of MCV1 coverage, Measles Incidence rate, Vaccine confidence, Refugee population, Peace Index, Climate index and HDI as Factor scores across WHO regions.** Factor components include Vaccinations: Measles Incidence Rate and Measles Vaccine coverage, Vaccine Confidence: Net Vaccine confidence percent, Conflict: Peace index score and Percent refugee origin population, Climate: Climate risk index, Education & Poverty: HDI index


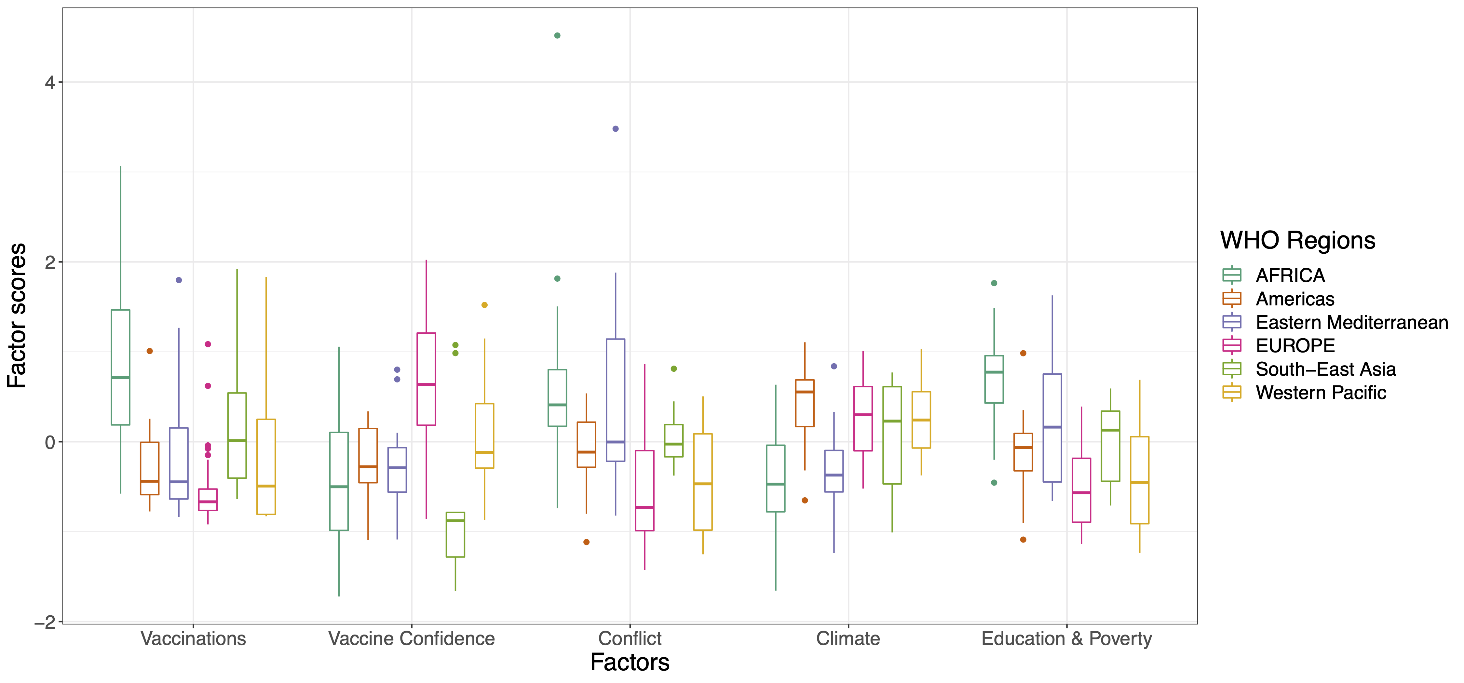

Supplement: S1 File — (DOCX) [file pone.0272784.s002.docx]
